# 4196 MICROBIAL COMPOSITION DEFINES PELVIC PAIN PHENOTYPES IN REPRODUCTIVE-AGE WOMEN

**DOI:** 10.1017/cts.2020.82

**Published:** 2020-07-29

**Authors:** A. Lenore Ackerman, Muhammed Umair Khalique, James E. Ackerman, Zhi Cheng, Karyn S. Eilber, Jennifer T. Anger, David M. Underhill

**Affiliations:** 1 David Geffen School of Medicine at UCLA; 2 Cedars-Sinai Medical Center

## Abstract

OBJECTIVES/GOALS: In young women, there is significant symptomatic overlap among lower urinary tract conditions, including bladder and pelvic pain, leading to misdiagnosis and delayed care. The epidemiology of pelvic pain suggests a microbial involvement, but previous studies have not definitively identified specific bacteria associated with pain diagnoses. METHODS/STUDY POPULATION: We examined urinary bacterial associations with specific symptom clusters, not diagnoses. Catheterized urinary samples were obtained from 78 pre-menopausal controls and cases with bladder and pelvic pain. 16S next-generation sequencing (NGS) characterized urinary microbial populations; validated questionnaires quantified symptom type and severity. *K* means unsupervised clustering analysis of NGS data assigned subjects to urotypes based on the urinary bacterial community state types. Quantitative PCR (qPCR) confirmed the NGS results and provided objective concentrations for critical taxa. Linear regression analysis confirmed the associations of bacterial concentrations and specific symptoms. RESULTS/ANTICIPATED RESULTS: In a pilot study of 35 reproductive-age women with a variety of complaints NGS revealed four urotypes that correlated with symptomatology. Isolated urgency incontinence was rare; the majority of subjects with symptoms complained of genitourinary pain. Bladder-specific pain (worse with filling, relieved by voiding) was associated with *Lactobacillus iners*. Asymptomatic patients almost universally had a non-iners, *Lactobacillus*-predominant microbiota. Vaginal and urethral pain unrelated to voiding correlated with increasing Enterobacteriaceae, primarily *Escherichia coli*. Detection of these species by qPCR in a validation population (n = 43) was highly predictive of each phenotype (P < 0.00001). Pathologic bacteria were associated with the severity of specific pain symptoms. DISCUSSION/SIGNIFICANCE OF IMPACT: Our results implicate a microbial role in genitourinary pain. We describe clinically-useful bacterial biomarkers for specific pelvic and bladder pain phenotypes. This objective, rapid, and inexpensive testing to classify bladder and pelvic pain would allow more accurate diagnosis and improve treatment. CONFLICT OF INTEREST DESCRIPTION: Dr. Anger is an expert witness for Boston Scientific. Dr. Eilber is an investigator and expert witness for Boston Scientific, an investigator for Aquinox, and a consultant for Boston Scientific and Allergan. Dr. Ackerman is an expert witness for Cynosure.

